# Triple Function of Synaptotagmin 7 Ensures Efficiency of High-Frequency Transmission at Central GABAergic Synapses

**DOI:** 10.1016/j.celrep.2017.10.122

**Published:** 2017-11-21

**Authors:** Chong Chen, Rachel Satterfield, Samuel M. Young, Peter Jonas

**Affiliations:** 1 IST Austria (Institute of Science and Technology Austria), Am Campus 1, A-3400 Klosterneuburg, Austria; 2 Max Planck Florida Institute for Neuroscience, Research Group Molecular Mechanisms of Synaptic Function, Jupiter, FL 33458, USA; 3 Department of Anatomy and Cell Biology, Department of Otolaryngology, Iowa Neuroscience Institute, Aging Mind Brain Initiative, University of Iowa, Iowa City, IA 52242, USA

## Abstract

Synaptotagmin 7 (Syt7) is thought to be a Ca^2+^ sensor that mediates asynchronous transmitter release and facilitation at synapses. However, Syt7 is strongly expressed in fast-spiking, parvalbumin-expressing GABAergic interneurons, and the output synapses of these neurons produce only minimal asynchronous release and show depression rather than facilitation. To resolve this apparent contradiction, we examined the effects of genetic elimination of Syt7 on synaptic transmission at the GABAergic basket cell (BC)-Purkinje cell (PC) synapse in cerebellum. Our results indicate that at the BC-PC synapse, Syt7 contributes to asynchronous release, pool replenishment, and facilitation. In combination, these three effects ensure efficient transmitter release during high-frequency activity and guarantee frequency independence of inhibition. Our results identify a distinct function of Syt7: ensuring the efficiency of high-frequency inhibitory synaptic transmission.

## Graphical abstract

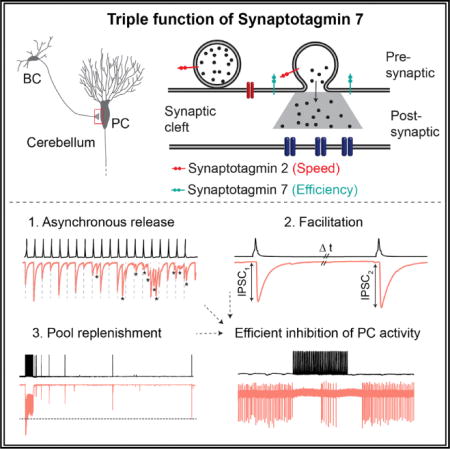

## INTRODUCTION

Synaptotagmins play a key role in Ca^2+^-dependent transmitter release (Südhof, 2002; Chapman, 2002; Koh and Bellen, 2003). The mammalian genome encodes 17 synaptotagmins, eight of which bind Ca^2+^ (Syt1, Syt2, Syt3, Syt5, Syt6, Syt7, Syt9, and Syt10; Südhof, 2002). Three of the synaptotagmins, Syt1, Syt2, and Syt9, have been demonstrated to operate as fast release sensors (Geppert et al., 1994; Xu et al., 2007; Kerr et al., 2008; Kochubey et al., 2016; Chen et al., 2017). However, the function of the remaining isoforms remains unclear. Syt7 is highly expressed in the brain, but its function is controversial (Chen and Jonas, 2017). In the zebrafish neuromuscular junction and in the young calyx of Held, Syt7 promotes asynchronous release during repetitive stimulation (Wen et al., 2010; Bacaj et al., 2013; Luo and Südhof, 2017). However, in cultured hippocampal synapses, Syt7 was suggested to promote replenishment of the releasable vesicle pool (Liu et al., 2014). Finally, in hippocampal and corticothalamic synapses in slices, Syt7 was recently proposed to operate as a Ca^2+^ sensor of synaptic facilitation (Jackman et al., 2016). How these divergent functions can be reconciled remains to be determined. One possibility is that Syt7 has different functions at different synapses.

Recent results indicate that Syt7 is highly expressed in inhibitory synapses. For example, Syt7 mRNA is abundant in fast-spiking, parvalbumin-expressing (PV^+^) basket cells in hippocampus and cerebellum (Kerr et al., 2008; Paul et al., 2012; Földy et al., 2016). However, the GABAergic output synapses of both cerebellar and hippocampal basket cells (BCs) show only minimal asynchronous transmitter release during and after action potential (AP) trains (Hefft and Jonas, 2005; Eggermann and Jonas, 2011; Arai and Jonas, 2014). Furthermore, the output synapses of these neurons exhibit synaptic depression rather than facilitation during repetitive stimulation (Kraushaar and Jonas, 2000; Eggermann and Jonas, 2011). Thus, the functional properties of synaptic transmission appear inconsistent with several of the proposed functions of Syt7 (Bacaj et al., 2013; Jackman et al., 2016). Finally, both cerebellar and hippocampal BC synapses show fast and temporally precise transmitter release (Hu et al., 2014). How Syt7, which shows slow Ca^2+^ binding and unbinding kinetics (Hui et al., 2005), would contribute to rapid signaling at these synapses remains unclear.

To examine the possible function of Syt7 in inhibitory synaptic transmission, we investigated the effects of deletion of the Syt7 gene at identified GABAergic BC-Purkinje cell (PC) synapses in cerebellar slices (Arai and Jonas, 2014). This synapse offers several technical advantages, including unique time and amplitude resolution, relative uniformity of the interneuron population, and accessibility to genetic manipulation (Arai and Jonas, 2014; Chen et al., 2017). Our results indicate that three apparently contradictory functions of Syt7 coexist at the same synapse. Through this triple function, Syt7 conveys efficient and frequency-independent transmitter release during repetitive activity. Finally, Syt7 enables the powerful control of PC activity by single BC synaptic inputs in cerebellar microcircuits.

## RESULTS

We examined the functional contribution of Syt7 to transmitter release at the GABAergic BC-PC synapse in the cerebellum, a synapse ideal for the biophysical analysis of GABAergic synaptic transmission (Eggermann and Jonas, 2011; Arai and Jonas, 2014; Chen et al., 2017). As a first step, we probed the expression of Syt7 (Figure S1). Immunolabeling with Syt7 antibodies revealed that Syt7 was highly expressed throughout the cerebellum. Immunoreactivity was largely abolished in *Syt7*^−/−^ mice, demonstrating the specificity of the labeling. Double labeling with GAD65 antibodies further suggested that Syt7 was strongly expressed in inhibitory terminals surrounding PC somata (Figure S1A). Furthermore, microarray analysis of mRNA expression in cerebellar BCs and stellate cells (Paul et al., 2012) suggested that, among all Ca^2+^-binding synaptotagmins, Syt7 was the most abundant isoform (Figure S1B). Thus, Syt7 is highly expressed in cerebellar BC presynaptic terminals.

**Figure 1:**
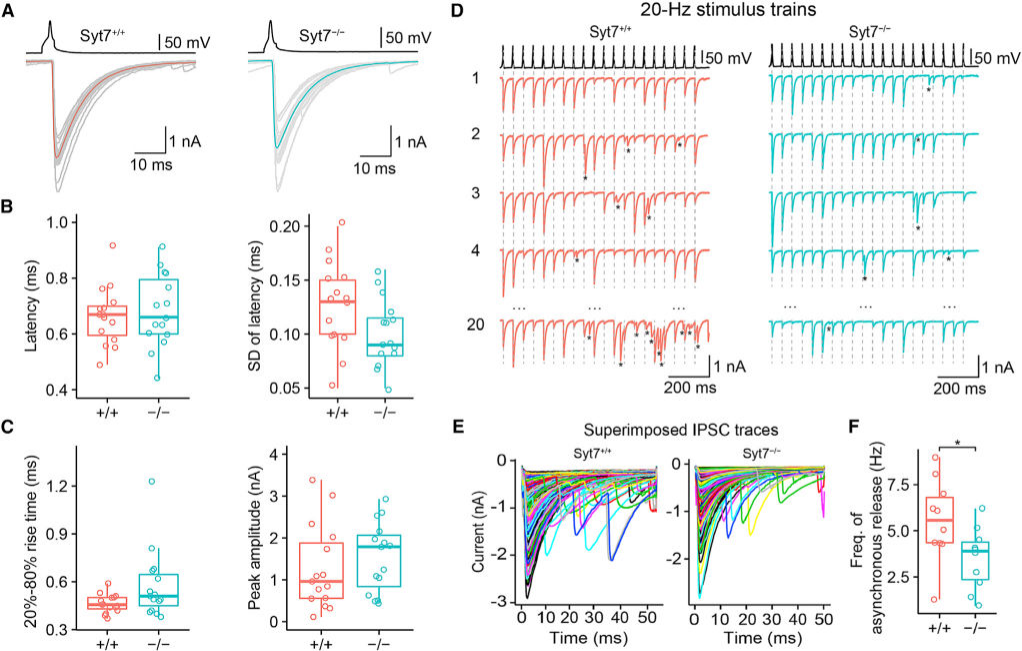
Syt7 Is a Ca^2+^ Sensor for Asynchronous, but Not Synchronous, Release at BC-PC Synapses (A) Evoked IPSCs in *Syt7*^+/+^ (left) and *Syt7*^−/−^ (right) mice. Top, presynaptic AP evoked by short current pulses in the presynaptic BC; bottom, IPSCs recorded in the synaptically connected PC (gray traces, individual sweeps; red and cyan traces, average IPSCs). (B and C) Boxplots of synaptic latency from the peak of the presynaptic AP to the onset of the IPSC (B, left), standard deviation of latency (B, right), 20%–80% rise time (C, left), and IPSC peak amplitude (C, right). Data from 15 pairs for *Syt7*^+/+^ and 15 pairs for *Syt7*^−/−^. (D) Synchronous and asynchronous release during 20-Hz stimulation trains in *Syt7*^+/+^ (left) and *Syt7*^−/−^ (right) mice. Asterisks represent asynchronous release events, and vertical dashed lines indicate the peaks of the presynaptic APs. (E) Synchronous and asynchronous IPSCs during 20-Hz stimulus trains. Left, superimposed IPSCs shown at expanded timescale (aligned to the peak of the presynaptic AP at t = 0). Individual traces were color coded to enhance visibility. (F) Boxplots of asynchronous release frequency. Asynchronous release was quantified in time intervals 15–50 ms after each presynaptic AP. The asterisk (*) indicates p = 0.034. Data from 10 pairs for *Syt7*^+/+^ and 10 pairs for *Syt7*^−/−^ mice. In boxplots (B, C, and F), horizontal lines represent median; boxes, quartiles; whiskers, most extreme data points ≤ 1.5 interquartile range from box edges; and single points, data from individual experiments.

To determine the functional contribution of Syt7 to GABAergic synaptic transmission, we compared cerebellar BC-PC synapses in wild-type (*Syt7*^+/+^) and knockout mice (*Syt7*^−/−^; Chakrabarti et al., 2003; Figure 1; Table S1). To evoke unitary inhibitory postsynaptic currents (IPSCs), we performed paired recordings in slices from 14- to 16-day-old mice (Eggermann and Jonas, 2011; Arai and Jonas, 2014; Chen et al., 2017). Genetic elimination of Syt7 did not change the basic properties of synaptic transmission following single APs (Figures 1A–1C; Table S1). For example, deletion of Syt7 did not alter the IPSC latency, the standard deviation of the latency as a measure for temporal precision of transmitter release, the 20%–80% rise time, the IPSC peak amplitude, and the IPSC decay time constant (p > 0.1 in all cases except SD, where p = 0.070; 15 pairs in *Syt7*^+/+^ and 15 pairs in *Syt7*^−/−^ slices). Additionally, genetic elimination of Syt7 did not change the amplitude or frequency of miniature IPSCs, demonstrating Syt7 does not contribute to spontaneous release (Figure S2; Table S1). These results confirm previous results that Syt7 does not contribute to synchronous transmitter release after single APs or spontaneous release in the absence of APs (Bacaj et al., 2013).

To test whether Syt7 contributes to asynchronous release during high-frequency stimulus trains (Wen et al., 2010; Bacaj et al., 2013; Luo and Südhof, 2017), we measured synchronous and asynchronous IPSCs during a 20-Hz repetitive stimulation (Arai and Jonas, 2014; Figures 1D–1F). The ideal recording conditions at the BC-PC synapse allowed us to perform direct counting of individual synaptic events. Asynchronous events were counted in a time window of 15–50 ms after each presynaptic AP. Genetic deletion of Syt7 significantly reduced the frequency of asynchronous release during the train from 5.57 ± 0.70 Hz in *Syt7*^+/+^ to 3.51 ± 0.52 Hz in *Syt7*^−/−^ synapses (10 pairs in each group; p = 0.034; Figure 1F). In conclusion, Syt7 contributed to the initiation of asynchronous release during high-frequency stimulus trains, although this function was less prominent at BC-PC synapses than at other previously examined synapses (Wen et al., 2010; Bacaj et al., 2013).

**Figure 2:**
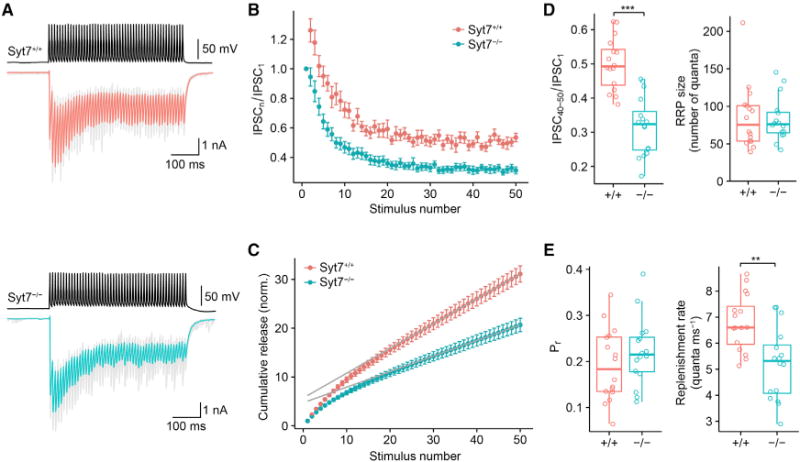
Syt7 Promotes Facilitation and Vesicle Pool Replenishment during Stimulus Trains at BC-PC Synapses (A) IPSCs evoked by a 100-Hz train of 50 APs for *Syt7*^+/+^ (top) and *Syt7*^−/−^ (bottom) mice. Upper traces, presynaptic APs evoked by brief current pulses; lower traces, IPSCs (gray traces, individual sweeps; red and cyan traces, average IPSCs). (B) Normalized IPSC peak amplitudes (IPSC_n_/IPSC_1_) plotted against stimulus number (n). Red circles, *Syt7*^+/+^; cyan circles, *Syt7*^−/−^ synapses. Data were obtained from 16 pairs in each group. (C) Quantitative analysis of pool size and refilling rate. IPSC peak amplitude was divided by IPSC_1_, averaged across cells, and cumulatively plotted against stimulus number. The last ten points were fit by linear regression. Size of the readily releasable pool (RRP) was determined from intersection of the regression line with the ordinate, whereas refilling rate was determined from the slope of the line. Release probability was quantified as the ratio of IPSC_1_ over pool size. (D and E) Boxplots for IPSC_40–50_/IPSC_1_ (D, left), RRP size (D, right), release probability (E, left), and replenishment rate (E, right). The triple asterisk (***) in (D) indicates p < 0.001, and the double asterisk (**) in (E) indicates p = 0.003.

To investigate whether Syt7 contributed to synaptic dynamics (Jackman et al., 2016) or vesicle pool replenishment (Liu et al., 2014), we applied 100-Hz stimulus trains, expected to maximally deplete the vesicle pool (Figure 2; Table S2). Plot of normalized IPSC amplitude against stimulus number revealed significant differences between *Syt7*^+/+^ and *Syt7*^−/−^ synapses (Figures 2A and 2B). In *Syt7*^+/+^ synapses, IPSCs showed a slight initial facilitation to 126.1% ± 5.8%, followed by depression to a steady-state amplitude of 53.0% ± 2.7% of the initial value (16 pairs). In marked contrast, in *Syt7*^−/−^ synapses, initial facilitation was abolished (94.5% ± 6.1%), and the steady-state IPSC amplitude was markedly reduced to 31.2% ± 2.3% (16 pairs, p < 0.001; Figure 2B). The effect of genetic deletion of Syt7 was rescued by helper-dependent adenovirus (HdAd)-mediated expression of Syt7, corroborating the validity of the genetic deletion approach (Figure S3). Interestingly, the steady-state transmission level after HdAd-mediated rescue in *Syt7*^−/−^ exceeded that in *Syt7*^+/+^ synapses, suggesting that expression levels of Syt7 after rescue were higher than endogenous levels (Figure S3). To determine the mechanisms underlying these changes, we computed the cumulative release, plotted it against stimulus number, and fit the data points for the last ten stimuli by linear regression (Schneggenburger et al., 1999; Figure 2C). The size of the releasable pool, determined by intersection of the regression line with the ordinate (Figure 2D), was not significantly different between *Syt7*^+/+^ and *Syt7*^−/−^ synapses (85 ± 11 vesicles versus 82 ± 7.0 vesicles; p = 0.88). In contrast, the replenishment rate, determined from the slope of the regression line, was significantly larger in *Syt7*^+/+^ than in *Syt7*^−/−^ synapses (6.79 ± 0.26 quanta ms^−1^ versus 5.21 ± 0.34 quanta ms^−1^; p = 0.003). Taken together, genetic deletion and rescue experiments suggest that Syt7 may regulate both facilitation and pool replenishment during high-frequency stimulation.

**Figure 3:**
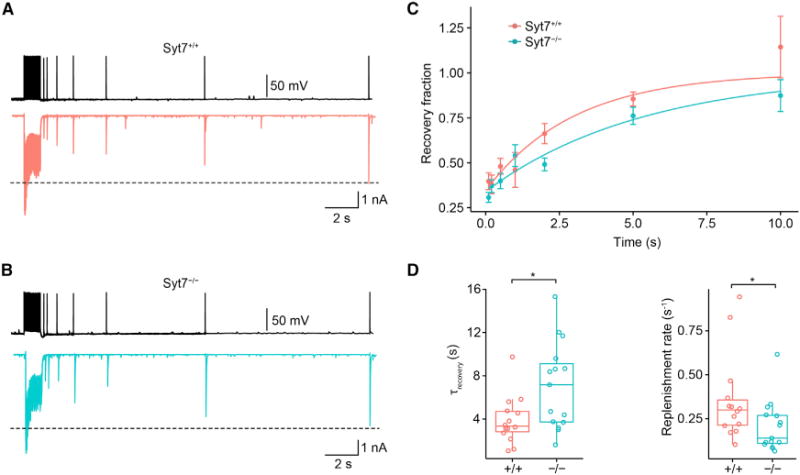
Syt7 Promotes Vesicle Pool Replenishment after Pool Depletion (A and B) IPSCs evoked by 100-Hz trains of 50 stimuli, followed by single test stimuli at variable time intervals in *Syt7*^+/+^ (A) and *Syt7*^−/−^ (B) synapses. Overlay of 7 traces; each trace represents the average from 5 individual sweeps. (C) Plot of peak amplitude of IPSC evoked by the test stimulus, normalized to the amplitude of the first IPSC in the preceding train. Continuous curves represent exponential functions fit to the data points (*Syt7*^+/+^, red; *Syt7*^−/−^, cyan). Data were obtained from 14 pairs for *Syt7*^+/+^ and 15 pairs for *Syt7*^−/−^. (D) Boxplots for recovery time constant (left) and corresponding replenishment rate (right). The asterisk (*) indicates p = 0.033 in both cases.

Next, we tested whether Syt7 affected the time course of vesicle pool refilling after a train of APs (Liu et al., 2014; Figure 3). To measure the time course of refilling, a 100-Hz train of 50 stimuli was applied to deplete the pool, followed by a single stimulus at various time points to probe the replenishment of the pool. In *Syt7*^+/+^ synapses, recovery from depression was fast, with a mean time constant of 3.89 ± 0.59 s (14 pairs; Figures 3C and 3D). In contrast, in *Syt7*^−/−^ synapses, recovery from depression was substantially prolonged, with a mean time constant of 7.05 ± 1.03 s (15 pairs; p = 0.033; Figures 3C and 3D). These results are consistent with the hypothesis that Syt7 promotes vesicle replenishment during and after high-frequency AP trains.

**Figure 4:**
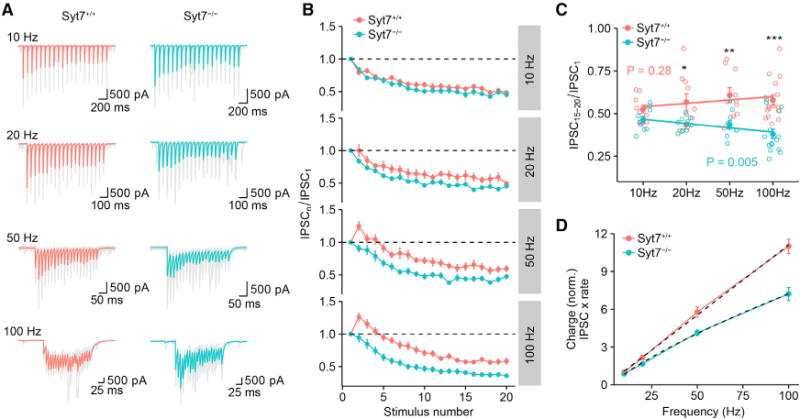
Syt7 Ensures Efficiency of High-Frequency Synaptic Transmission and Linearizes the Input-Output Relation of BC-PC Synapses (A) IPSCs evoked by trains of 20 APs at different frequencies for *Syt7*^+/+^ (left) and *Syt7*^−/−^ synapses (right; gray traces, individual sweeps; red and cyan traces, average IPSCs). (B) Normalized IPSC peak amplitudes (IPSC_n_/IPSC_1_), plotted against stimulus number (n). Red circles, *Syt7*^+/+^; cyan circles, *Syt7*^−/−^. Data were obtained from 10/10 (10 Hz), 10/10 (20 Hz), 10/10 (50 Hz), and 16/16 pairs (100 Hz). (C) Plot of normalized steady-state IPSC amplitude, as quantified by IPSC_15–20_/IPSC_1_, against stimulus frequency. Open circles represent data from individual experiments, filled circles indicate mean ± SEM. Lines represent results from linear regression analysis (Spearman ρ = 0.16, p = 0.28 for *Syt7*^+/+^ synapses; ρ = −0.41, p = 0.005 for *Syt7*^−/−^ synapses). *, **, and *** indicate p = 0.043, 0.003, and < 0.001, respectively. Note that normalized steady-state IPSC amplitude does not show significant frequency dependence in *Syt7*^+/+^ synapses but acquires marked frequency dependence in *Syt7*^−/−^ synapses. (D) Plot of normalized steady-state charge, as quantified by IPSC peak amplitude multiplied with stimulation frequency. Dashed lines represent the results from fit with a linear function (*Syt7*^+/+^) or a power function (*Syt7*^−/−^). In *Syt7*^+/+^ synapses, inhibitory charge was linearly dependent on stimulation frequency. In contrast, in *Syt7*^−/−^ synapses, the dependence was sublinear.

To examine the frequency dependence of the effects of Syt7 deletion during repetitive stimulation (Bagnall et al., 2008; Turecek et al., 2016), we examined IPSCs evoked by 10–100 Hz stimulus trains (Figure 4; Table S2). Whereas for 10 Hz the extent of steady-state depression was identical between *Syt7*^+/+^ and *Syt7*^−/−^ synapses, the differences between *Syt7*^+/+^ and *Syt7*^−/−^ synapses progressively increased for frequencies of 20–100 Hz (p = 0.09, 0.04, 0.003, and <0.001; 10/10, 10/10, 10/10, and 16/16 pairs; Figure 4C). Linear regression analysis revealed that the ratio IPSC_15–20_/IPSC_1_ was not significantly frequency dependent in *Syt7*^+/+^ synapses (p = 0.28) but highly frequency dependent in *Syt7*^−/−^ synapses (p = 0.005; Figure 4C). Thus, Syt7 contributed to the frequency independence of transmitter release at BC-PC synapses during repetitive stimulation (Turecek et al., 2016). To further analyze the functional significance of this property of BC-PC synapses, we quantified the frequency dependence of the inhibitory synaptic charge (Turecek et al., 2016). In *Syt7*^+/+^ synapses, the charge-frequency relation was linear, whereas in *Syt7*^−/−^ synapses, a marked sublinearity was apparent (Figure 4D). Thus, Syt7 linearized input-output conversion in BC-PC synapses.

Our results indicate that Syt7 enhances transmitter release at cerebellar BC-PC synapses during repetitive firing. To find out whether this is a general mechanism that also operates at output synapses of fast-spiking interneurons in other brain areas, we examined the effects of genetic elimination of Syt7 in hippocampal basket cell to granule cell (BC-GC) synapses (Kraushaar and Jonas, 2000; Hefft and Jonas, 2005; Figure S4). Deletion of Syt7 significantly increased the extent of depression during trains of 20 APs at 50 Hz (IPSC_15–20_/IPSC_1_ = 0.22 ± 0.01 in *Syt7*^+/+^ versus 0.13 ± 0.02 in *Syt7*^−/−^ synapses; p = 0.02; 5 pairs in each group; Figure S4). Thus, Syt7 promoted high-frequency transmission in hippocampal BC-GC synapses, although the effect was smaller than in cerebellar BC-PC synapses. Therefore, the function of Syt7 is conserved in at least two different types of rapidly signaling GABAergic synapses.

**Figure 5:**
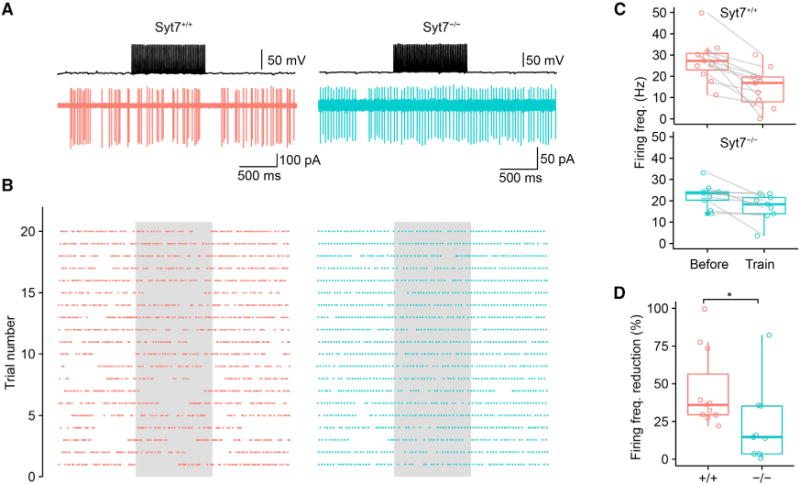
Syt7 Ensures Efficient Suppression of PC Activity by Unitary Inhibitory Synaptic Inputs (A) Simultaneous paired recording from a presynaptic BC (whole-cell current clamp configuration, upper trace) and a postsynaptic PC (noninvasive cell-attached voltage-clamp configuration, lower trace) for a *Syt7*^+/+^ (left) or *Syt7*^−/−^ synapse (right). Upper, presynaptic APs; lower, postsynaptic action currents during the same period. A 1 s, 50-Hz stimulus train was applied to the presynaptic BC. Note that activation of the unitary BC input suppressed spontaneous activity in PCs, as indicated by a marked reduction in the frequency of action currents. (B) Raster plot from 20 single trials for a *Syt7*^+/+^ (left) or *Syt7*^−/−^ synapse (right). Each point represents an action current in the PC. Gray area indicates time period of BC stimulation. Trial 5 corresponds to traces shown in (A). (C) Boxplots of firing frequency in PCs before and during train stimulation of BCs. Lines connect data from the same experiment. Top, *Syt7*^+/+^; bottom, *Syt7*^−/−^. (D) Boxplot of reduction of firing frequency in PCs by train stimulation of BCs for *Syt7*^+/+^ (red) and *Syt7*^−/−^ synapses (cyan). The asterisk (*) indicates p = 0.02. In boxplots in (C) and (D), horizontal lines represent median; boxes, quartiles; whiskers, most extreme data points ≤ 1.5 interquartile range from box edges; and single points, data from individual experiments.

Our results demonstrate that Syt7 promotes high-frequency transmission at GABAergic interneuron output synapses. Are these effects relevant for the function of principal neuron-interneuron microcircuits? To address this question, we performed paired recordings between cerebellar BCs in the whole-cell current-clamp configuration and PCs in the noninvasive cell-attached configuration (Figure 5). Presynaptic BCs were activated with a 1 s, 50-Hz stimulation, intended to mimic a high activity level of these neurons under *in vivo* conditions (Jelitai et al., 2016). In the absence of presynaptic stimulation, PCs showed spontaneous firing in both *Syt7*^+/+^ and *Syt7*^−/−^ mice (Figures 5A–5C). In *Syt7*^+/+^ mice, a 1 s, 50-Hz stimulation of a single presynaptic BC caused a reduction of action current frequency in PCs by 45.8% ± 7.7% (11 pairs; Figures 5C and 5D), demonstrating the efficacy of unitary inhibitory synaptic inputs in regulating PC spiking (Häusser and Clark, 1997). In contrast, in slices from *Syt7*^−/−^ mice, high-frequency stimulation of a single presynaptic BC reduced PC activity by only 22.8% ± 8.5% (p = 0.028; 9 pairs; Figures 5C and 5D). Taken together, these results indicate that Syt7 plays a key role to ensure the efficient regulation of PC activity by unitary GABAergic inputs in cerebellar microcircuits.

## DISCUSSION

The role of Syt7 in transmitter release at central synapses has been controversial. Several functions have been proposed, including a trigger function for asynchronous release during stimulus trains (Wen et al., 2010; Bacaj et al., 2013; Luo and Südhof, 2017), an acceleration of vesicle pool replenishment (Liu et al., 2014), and a function as a facilitation sensor (Jackman et al., 2016; Jackman and Regehr, 2017). Our results show that these functions are not mutually exclusive but coexist at single BC-PC synapses. In combination, these mechanisms ensure efficiency and frequency independence of inhibitory synaptic transmission at GABAergic synapses. Furthermore, our results suggest that the function of Syt7 is conserved across output synapses of fast-spiking, GABAergic interneurons, at least between cerebellum and hippocampus. Finally, our experiments show that Syt7, at the network level, enables the powerful control of PC activity by single BC synaptic inputs. As PCs represent the sole output from the cerebellum, this puts Syt7 into a strategic position to regulate the information flow in this motor circuit. Although our conclusions are largely based on a knockout approach and therefore require careful interpretation, the results from the rescue experiments are fully consistent with our hypotheses.

How does a single protein, Syt7, generate three divergent effects at BC-PC synapses? Several models of synaptotagmin action suggest that a single primary mechanism may underlie the three effects. It has been proposed that synaptotagmins reduce the energy level of membrane fusion intermediates (Jackman and Regehr, 2017). In this model, both Syt7 and fast synaptotagmins (Syt1, Syt2, or Syt9; Xu et al., 2007) would reduce the activation energy of fusion, Syt7 by a small amount and Syt1, Syt2, or Syt9 by a larger amount. In consequence, Syt7 in isolation would trigger asynchronous release but, in conjunction with fast synaptotagmins, would promote facilitation, because additive effects on energy barriers cause supralinear effects on vesicle fusion rate (Schotten et al., 2015). In parallel, Syt7 may reduce the activation energy of docking or priming, thus explaining acceleration of vesicle replenishment.

Syt7 may also primarily act as a Ca^2+^ sensor of activity-dependent replenishment (Liu et al., 2014) by accelerating vesicle tethering, docking, or priming (Imig et al., 2014; Bacaj et al., 2015). The subcellular localization of Syt7, in plasma membrane and intracellular organelles (Sugita et al., 2001; Liu et al., 2014; Li et al., 2017), might be consistent with these hypotheses. In this model, Syt7 may promote filling of the vesicular pools, leading to an increase in docking site occupancy (Pulido et al., 2015). Accordingly, Syt7 would also enhance both asynchronous release and facilitation. Alternatively, Syt7 could affect replenishment by accelerating endocytosis (Poskanzer et al., 2003; Hosoi et al., 2009). Although the kinetics of replenishment may be difficult to reconcile with clathrin-mediated slow endocytosis, it might be compatible with clathrin-independent fast endocytosis (Delvendahl et al., 2016).

Finally, the interrelation of the three effects might be explained by heterogeneity of the vesicular pool. Syt7 could trigger release from a separate slow pool of vesicles (Schonn et al., 2008), whereas Syt1, Syt2, or Syt9 would trigger release from a fast pool (Turecek et al., 2016; Chen et al., 2017). If the fast pool shows high release probability, slow recovery, and depression and the slow pool has low release probability, fast recovery, and facilitation (Sakaba and Neher, 2001; Turecek et al., 2016), Syt7 deletion may slow recovery and reduce facilitation in parallel, as observed experimentally.

It is well established that Syt7 operates as a Ca^2+^ sensor for asynchronous release at several synapses (Wen et al., 2010; Bacaj et al., 2013; Luo and Südhof, 2017). Our results are consistent with this function but show that it plays a relatively minor role at the BC-PC synapse. Why is this the case? One important factor that determines the rate of asynchronous release is the coupling distance between Ca^2+^ channels and release sensors (Eggermann et al., 2011). In both cerebellar BC-PC synapses and hippocampal BC-GC synapses, tight “nanodomain” coupling governs transmitter release from active zones (Bucurenciu et al., 2008; Arai and Jonas, 2014). Tight coupling is expected to reduce the relative amount of asynchronous release (Bucurenciu et al., 2008; Eggermann et al., 2011). Thus, asynchronous release is small in wild-type synapses, and additional effects of Syt7 elimination are expected to be minimal. Likewise, tight coupling will prevent certain forms of facilitation; e.g., facilitation by saturation of presynaptic buffers (Felmy et al., 2003; Vyleta and Jonas, 2014). In contrast, at synapses with looser coupling (such as hippocampal synapses in culture or the young calyx of Held), asynchronous release and facilitation will be more pronounced, and the effects of Syt7 deletion will be more prominent (Bacaj et al., 2013; Luo and Südhof, 2017).

Our results have major implications for the stability of inhibition in neuronal networks *in vivo*. Fast-spiking, PV^+^ interneurons in different brain regions generate APs at high frequency. For example, PV^+^ interneurons in the cerebellum fire APs with a frequency of up to 100 Hz during active movement (Jelitai et al., 2016), and PV^+^ interneurons in the hippocampal CA1 region generate high-frequency trains of spikes during sharp-wave ripples (Lapray et al., 2012). Under these conditions, PV^+^ interneurons need to generate stable and reliable inhibitory output signals. Our results suggest that the expression of Syt7 plays a critical role in maintaining the efficacy of inhibitory synaptic transmission at these synapses. Thus, whereas the fast Ca^2+^ sensor Syt2 is responsible for the speed and temporal precision of transmitter release (Kerr et al., 2008; Chen et al., 2017), Syt7 plays a critical role in maintaining the efficacy during high-frequency synaptic transmission at cerebellar and hippocampal GABAergic synapses. Whether these results generalize to other fast-signaling synapses throughout the brain remains to be determined.

## EXPERIMENTAL PROCEDURES

### Contact for Reagent and Resource Sharing

As Lead Contact, Peter Jonas is responsible for all reagent and resource requests. Please contact Peter Jonas at peter.jonas@ist.ac.at with requests and inquiries.

### Experimental Model and Subject Details

Experiments on C57BL/6 wild-type and mutant mice were performed in strict accordance with institutional, national, and European guidelines for animal experimentation and were approved by the Bundesministerium für Wissenschaft, Forschung und Wirtschaft of Austria (A. Haslinger, Vienna; BMWFW-66.018/0007-WF/II/3b/2014; BMWF-66.018/0010-WF/V/3b/2015; BMWFW-66.018/0020-WF/V/3b/2016).

### Immunohistochemistry, Cerebellar Slice Preparation, Paired Recordings, Production and Injection of Adenoviral Expression Vectors, Data Acquisition, and Analysis

Further details and an outline of resources used in this work can be found in the Supplemental Experimental Procedures.

### Quantification and Statistical Analysis

All values were reported as mean ± SEM. Statistical significance was tested using nonparametric Kruskal-Wallis and two-sided Wilcoxon rank-sum tests in R. Differences with p < 0.05 were considered significant. In figures, a single asterisk (*), double asterisks (**), and triple asterisks (***) indicate p < 0.05, p < 0.01, and p < 0.001, respectively. In boxplots, horizontal lines represent medians, boxes represent quartiles, whiskers represent the most extreme data points ≤ 1.5 interquartile range from box edges, and single points represent data from individual experiments. In total, data were obtained from 165 cerebellar BC-PC pairs (80 from *Syt7*^+/+^ and 85 from *Syt7*^−/−^), 19 PC recordings (10 from *Syt7*^+/+^ and 9 from *Syt7*^−/−^), and 10 hippocampal BC-GC pairs (5 from *Syt7*^+/+^ and 5 from *Syt7*^−/−^).

## Supplementary Material

supplemental

## Highlights

- Syt7 contributes to asynchronous release, facilitation, and replenishment
- Syt7 conveys efficient and frequency-independent release during repetitive activity
- Syt7 enables the powerful control of PC activity by single BC synaptic inputs
- Syt7 has similar functions at cerebellar and hippocampal BC synapses
