# Supplementary material for: Triple Function of Synaptotagmin 7 Ensures Efficiency of High-Frequency Transmission at Central GABAergic Synapses
Source: Cell Rep. Author manuscript; Available in PMC 2018 Mar 22. (PMC5863544; doi:10.1016/j.celrep.2017.10.122)
Supplement: supplemental [file NIHMS947306-supplement-supplemental.pdf]

Cell Reports, Volume *21*

Supplemental Information

Triple Function of Synaptotagmin 7 Ensures Efficiency of High-Frequency Transmission at Central GABAergic Synapses

Chong Chen, Rachel Satterfield, Samuel M. Young, Jr., and Peter Jonas

SUPPLEMENTAL EXPERIMENTAL PROCEDURES

Immunohistochemistry

14- to 16-day-old mice of either sex were transcardially perfused, using 4% paraformaldehyde in 100 mM phosphate buffer (PB; pH 7.35) for fixation. Brains were dissected out and post-fixed in 4% paraformaldehyde for ~24 h. 50 µm-thick slices were cut from the cerebellar vermis using a VT1200S vibratome (Leica Microsystems). After washing with 0.1 M PB, slices were incubated with 10% normal goat serum (NGS) for 1 h and subsequently with primary antibodies against Syt7 (polyclonal rabbit; Synaptic Systems, 105173; 1:200) and GAD65 (polyclonal guinea pig; Synaptic Systems, 198104; 1:500) both in PB containing 5% NGS and 0.5% Triton X-100 overnight. After washing, slices were incubated with specific secondary antibodies (Alexa Fluor 647-conjugated anti-rabbit secondary antibody; Alexa Fluor 488-conjugated anti-guinea pig secondary antibody; Thermo Fisher Scientific; 1:1000 for both) with PB containing 5% NGS and 0.3% TritonX-100 overnight. After washing, slices were embedded in Prolong Antifade and examined under a TCS SP5 II confocal microscope (Leica Microsystems).

Cerebellar slice preparation

C57BL/6 Syt7 knockout mice ($Syt7^{-/-}$), in which a stop codon was inserted in exon 4, were obtained from Jackson Labs (stock number 004950; generated by Norma Andrews; $Syt7^{tm1Nan}$; Chakrabati et al., 2003). All experiments were performed on littermate offspring from homozygous and heterozygous matings, with knockout mice being homozygous for the deletion allele ($Syt7^{-/-}$) and wild-type animals homozygous for the wild-type allele ($Syt7^{+/+}$). Previous work showed that deletion of Syt7 has no detectable effects on the expression of several other synaptic proteins (Maximov et al., 2008; Bacaj et al., 2013). Slices were cut from the vermis of the cerebellum of 14- to 16-day-old mice of either sex. In all experiments, genotypes were determined by polymerase chain reaction analysis. After decapitation, the brain was rapidly dissected out and immersed in ice-cold slicing solution containing: 87 mM NaCl, 25 mM $NaHCO_3$, 2.5 mM KCl, 1.25 mM NaH_2PO_4, 10 mM D-glucose, 75 mM sucrose, 0.5 mM $CaCl_2$, and 7 mM $MgCl_2$, (pH 7.4 in 95% O_2 / 5% CO_2, ~325 mOsm). Parasagittal 300-µm-thick cerebellar slices were cut using a VT1200 vibratome (Leica Microsystems). In a subset of experiments (**Figure S4**), slices were cut from the hippocampus of 14- to 16-day-old mice, as previously described (Kraushaar and Jonas, 2000; Hefft and Jonas,

2005). After ~20 min incubation at ~35ºC, the slices were stored at room temperature. Slices were used for maximally 5 hours after dissection. Experiments were performed at 21–24°C.

Paired recordings

During experiments, slices were superfused with a physiological extracellular solution containing: 125 mM NaCl, 2.5 mM KCl, 25 mM $NaHCO_3$, 1.25 mM NaH_2PO_4, 25 mM D-glucose, 2 mM $CaCl_2$, and 1 mM $MgCl_2$ (pH 7.4 in 95% O_2 / 5% CO_2, ~325 mOsm). Paired recordings from synaptically connected BCs and PCs were performed from lobule V and VI as previously described (Caillard et al., 2000; Sakaba, 2008; Eggermann and Jonas, 2012; Arai and Jonas, 2014; Chen et al., 2017). Intracellular solution used for the presynaptic BCs contained: 125 mM K-gluconate, 20 mM KCl, 0.1 mM EGTA, 10 mM phosphocreatine, 2 mM $MgCl_2$, 2 mM ATP, 0.4 mM GTP, and 10 mM HEPES (pH adjusted to 7.28 with KOH, ~310 mOsm); 0.2% biocytin was added in a subset of recordings. BCs were identified by the location of the soma in the inner molecular layer close to the PC layer under experimental conditions and by the formation for basket-like arborizations and Pinceau terminations around PC somata in biocytin-labeled material (Eggermann and Jonas, 2012; Arai and Jonas, 2014; Chen et al., 2017). The presynaptic pipette resistance was 8–10 MΩ. BCs were recorded under current-clamp conditions. A holding current of ~−50 pA was injected to maintain the membrane potential at ~−65 mV and to avoid spontaneous AP generation. To evoke presynaptic APs, single pulses or trains of 20 or 50 pulses at 10–100 Hz (400 pA, 4 ms) were injected into the presynaptic BC every 4 s or 20 s, respectively.

Intracellular solution for postsynaptic PCs contained: 140 mM KCl, 10 mM EGTA, 2 mM $MgCl_2$, 2 mM ATP, 10 mM HEPES, and 2 mM QX-314 (pH adjusted to 7.28 with KOH, ~313 mOsm). To achieve the lowest possible postsynaptic series resistance, large tip-sized patch pipettes were fabricated from leaded glass (PG10165-4, WPI) using a horizontal micropipette puller (P-97, Sutter Instrument). To reduce pipette capacitance, pipettes were coated with dental wax. The postsynaptic pipette resistance was 0.8–1.5 MΩ, resulting in a series resistance of 3–8 MΩ. Experiments in which series resistance changed by > 2 MΩ were discarded. PCs were recorded in the voltage-clamp configuration with a holding potential of −70 mV. For monitoring series and input resistance, 5-mV, 100-ms hyperpolarizing test pulses were applied after the IPSCs had decayed to baseline. For recording of miniature IPSCs (**Figure**

S2), synaptic events were examined in pharmacological isolation in the presence of 1 μM tetrodotoxin (TTX), 10 μM 6-cyano-7-nitroquinoxaline-2,3-dione (CNQX), and 20 μM D-2-amino-5-phosphonopentanoic acid (D-AP5) at −70 mV. For cell-attached recordings (**Figure 5**), recording pipettes were filled with intracellular solution (without QX-314), and pipette potential was adjusted to keep the holding current at ~0. In a subset of experiments (**Figure S4**), recordings were made from hippocampal BC–GC synapses, as previously described (Kraushaar and Jonas, 2000; Hefft and Jonas, 2005).

Production and injection of adenoviral expression vectors

Synaptotagmin 7 cDNA (alpha splice variant, *Mus musculus* (NP_061271); Sugita et al., 2001) was codon-optimized for expression in mouse (GeneArt) and then cloned into the EcoRI and NotI sites of the synapsin expression cassette (Montesinos et al., 2011; Montesinos et al., 2016; Chen et al., 2017). This cassette included the 470 bp human synapsin (hsyn) promoter, the minute virus of mice (mvm) intron, and the bovine growth hormone (BGH) polyA. Subsequently, the expression cassette was cloned into the AscI site of pC4HSU28. This version of pC4HSU28 was modified to also contain a separate neurospecific EGFP expression cassette driven by the 470 bp hsyn promoter.

HdAds were produced by first digesting the pHdAd with PmeI to linearize and expose the ends of the 5’ and 3’ inverted terminal repeats. Transfection of the pHdAd was performed using 116 producer cells, a modified HEK293 line expressing high levels of Cre recombinase, and a 4-kbp adenoviral genome fragment that encodes for the E1A/E1B gene. HdAd was serially amplified in 5 consecutive passages. Each successive passage was performed after cytopathic effect (CPE) occurred and cell lysates were subjected to 3 freeze-thaw cycles to lyse cells and thereby release the viral particles. HdAd was stored at −80°C in storage buffer containing 10 mM HEPES, 250 mM sucrose, and 1 mM $MgCl_2$ at pH 7.4.

Syt7$^{-/-}$ mice, at postnatal day (P) 3 to 6, were anesthetized using isoflurane (5% for induction and ~3% during the injection procedure; Forane®, Abbott) combined with meloxicam (1 mg kg^{-1}, Boehringer) for analgesia. Meloxicam was given 2 hours before surgery for pre-operative analgesia and repeated twice 24 h after the previous injection for post-operative analgesia. After sufficient sedation, mice were put on a stereotaxic apparatus and head-fixed with ear bars. The skin was cut, the skull was exposed, and

a small hole was made with a needle in the region over the cerebellum. 1 μl adenovirus (~10^9 vp) was injected into the vermis of the cerebellum at a depth of ~600 μm from the endocranium. After virus injection, pups were returned to their home cages for recovery. Recordings were made at P14 to 16. Infected cerebellar BCs were identified by EGFP fluorescence, using epifluorescence during experiments and confocal imaging for subsequent documentation.

Data acquisition and analysis

Data were acquired with a Multiclamp 700B amplifier (Axon Instruments), low-pass filtered at 10 kHz, and sampled at 20 kHz using a CED power1401 interface (Cambridge Electronic Design). Stimulus generation and data acquisition were performed using custom-made software (FPulse version 3.33, Ulrich Fröbe, University of Freiburg) running under Igor Pro 6.22 (WaveMetrics). Data were analyzed using Stimfit 0.14.9 (https://github.com/neurodroid/stimfit; Guzman et al., 2014), R 3.4.1 (the R project for statistical computing), and Mathematica 11.0 (Wolfram Research). Synaptic latency of monosynaptic IPSCs was measured from the peak of the presynaptic AP to the IPSC onset. IPSC decay time constant was determined by fitting the decay phase of an average IPSC trace. To quantify multiple-pulse depression, traces were averaged and the amplitude of each IPSC in a train was measured from the baseline directly preceding the rising phase. Asynchronous IPSCs during train stimulation (**Figure 1**) and miniature IPSCs in the presence of TTX (**Figure S2**) were detected using a template matching algorithm and verified by visual inspection (Pernía-Andrade et al., 2012). Asynchronous release rate was quantified in a time interval 15–50 ms after the peak of each presynaptic AP.

For analysis of vesicular pool size and refilling rate, IPSC amplitudes during a 100-Hz train of 50 stimuli were examined. 100-Hz stimulation was used to maximize the extent of depression. IPSC values were normalized by $IPSC_1$, averaged across cells, and cumulatively plotted against stimulus number. The last ten data points were fit by linear regression. The size of the readily releasable pool (RRP) was determined from intersection of the regression line with the ordinate, while refilling rate was determined from the slope (Schneggenburger et al., 1999). The RRP estimate represents “pool decrement” rather than absolute pool size; the true pool size may be larger than the estimate (Neher, 2015). To obtain absolute numbers of RRP size and refilling rate, estimated values were multiplied by the quantal content of $IPSC_1$. Quantal

size was determined as the mean peak amplitude of miniature IPSCs in each group (**Figure S2**). Control experiments in the presence of 300 μM of the low-affinity competitive $GABA_A$ receptor antagonist (1,2,5,6-tetrahydropyridine-4-yl)-methylphosphinic acid (TPMPA) and the $GABA_B$ receptor antagonist CGP 55845 (both Tocris) gave similar results, indicating that the analysis of pool size and refilling rate were not confounded by desensitization or saturation of postsynaptic receptors (Jones et al., 2001; Sakaba, 2008; Arai and Jonas, 2014; Chen et al., 2017).

Resource Table

REAGENT or RESOURCE	SOURCE	IDENTIFIER
**Antibodies**		
Polyclonal guinea pig anti-GAD65	Synaptic Systems	Cat. No.198104
Polyclonal rabbit anti-Syt7	Synaptic Systems	Cat. No. 105173
Goat anti-rabbit, Alexa-647	Thermo Fisher Scientific	Cat. No. A21244
Goat anti-guinea pig, Alexa-488	Thermo Fisher Scientific	Cat. No. A11703
**Bacterial and Virus Strains**		
HdAd-hsyn-syt7-EGFP	Prof. Samuel M. Young Jr.	N/A
**Chemicals, Peptides, and Recombinant Proteins**		
NaCl	VWR	1.06404
$NaHCO_3$	VWR	1.06329
KCl	VWR	1.049.360
NaH_2PO_4	VWR	1.06346
D-glucose	VWR	1.08342
sucrose	Sigma	16104
$MgCl_2$	Sigma	31413
$CaCl_2$	VWR	1.02382.0250
K-gluconate	Sigma	G4500
EGTA	Sigma	E0396
Phosphocreatine	Sigma	P7936
HEPES	Sigma	H3375
ATP	Sigma	A8937
GTP	Sigma	G8877
TTX	Tocris	BN0
CNQX	Tocris	BN0154 50
D-AP5	Tocris	0106
TPMPA	Tocris	1040
CGP 55845	Tocris	1248
**Deposited Data**		
Raw Microarray data	GEO (Paul et al., 2012)	GSE37055
**Experimental Models: Organisms/Strains**		
Mouse: $Syt7^{tm1Nan/J}$	The Jackson Laboratory	Stock No:004950
**Oligonucleotides**		
Syt7 primers	The Jackson Laboratory<br>Sigma	oIMR3347,<br>oIMR3348,<br>oIMR6916,<br>oIMR6917

**Recombinant DNA**		
Plasmid: Syt7	GeneArt	N/A
**Software and Algorithms**		
R 3.4.1 and Rstudio 1.0.153	The R Foundation	https://www.r-project.org/; https://www.rstudio.com/
Fiji 1.0	Schindelin et al., 2012	https://fiji.sc
Mathematica 11.0	Wolfram Research	http://www.wolfram.com/mathematica
Stimfit 0.14.9	Guzman et al., 2014	https://github.com/neurodroid/stimfit
Igor Pro 6.22	WaveMetrics	https://www.wavemetrics.com
FPulse 3.33	Ulrich Fröbe, University of Freiburg	N/A
**Patch clamp glass**		
Presynaptic pipette	Hilgenberg	1807524
Postsynaptic pipette	WPI	PG 10165-4

Supplemental References

Caillard, O., Moreno, H., Schwaller, B., Llano, I., Celio, M.R., and Marty, A. (2000). Role of the calcium-binding protein parvalbumin in short-term synaptic plasticity. Proc. Natl. Acad. Sci. USA *97*, 13372–13377.

Guzman, S.J., Schlögl, A., and Schmidt-Hieber, C. (2014). Stimfit: quantifying electrophysiological data with Python. Front. Neuroinform. *8*, 16.

Jones, M.V., Jonas, P., Sahara, Y., and Westbrook, G.L. (2001). Microscopic kinetics and energetics distinguish $GABA_A$ receptor agonists from antagonists. Biophys. J. *81*, 2660–2670.

Maximov, A., Lao, Y., Li, H., Chen, X., Rizo, J., Sørensen, J.B., and Südhof, T.C. (2008). Genetic analysis of synaptotagmin-7 function in synaptic vesicle exocytosis. Proc. Natl. Acad. Sci. USA *105*, 3986–3991.

Montesinos, M.S., Chen, Z., and Young, S.M. Jr. (2011). pUNISHER: a high-level expression cassette for use with recombinant viral vectors for rapid and long term in vivo neuronal expression in the CNS. J. Neurophysiol. *106*, 3230–3244.

Montesinos, M.S., Satterfield, R., and Young, S.M. Jr. (2016). Helper-dependent adenoviral vectors and their use for neuroscience applications. Methods Mol. Biol. *1474*, 73–90.

Neher, E. (2015). Merits and limitations of vesicle pool models in view of heterogeneous populations of synaptic vesicles. Neuron *87*, 1131–1142.

Pernía-Andrade, A.J., Goswami, S.P., Stickler, Y., Fröbe, U., Schlögl, A., and Jonas, P. (2012). A deconvolution-based method with high sensitivity and temporal resolution for detection of spontaneous synaptic currents in vitro and in vivo. Biophys. J. *103*, 1429–1439.

Sakaba, T. (2008). Two Ca^{2+}-dependent steps controlling synaptic vesicle fusion and replenishment at the cerebellar basket cell terminal. Neuron *57*, 406–419.

Schindelin, J., Arganda-Carreras, I., Frise, E., Kaynig, V., Longair, M., Pietzsch, T., Preibisch, S., Rueden, C., Saalfeld, S., Schmid, B., Tinevez, J.Y., White, D.J., Hartenstein, V., Eliceiri, K., Tomancak, P., and Cardona, A. (2012). Fiji: an open-source platform for biological-image analysis. Nat. Methods *9*, 676–682.

Figure S1. Syt7 is highly expressed in presynaptic terminals of cerebellar BCs, related to Figures 1–5

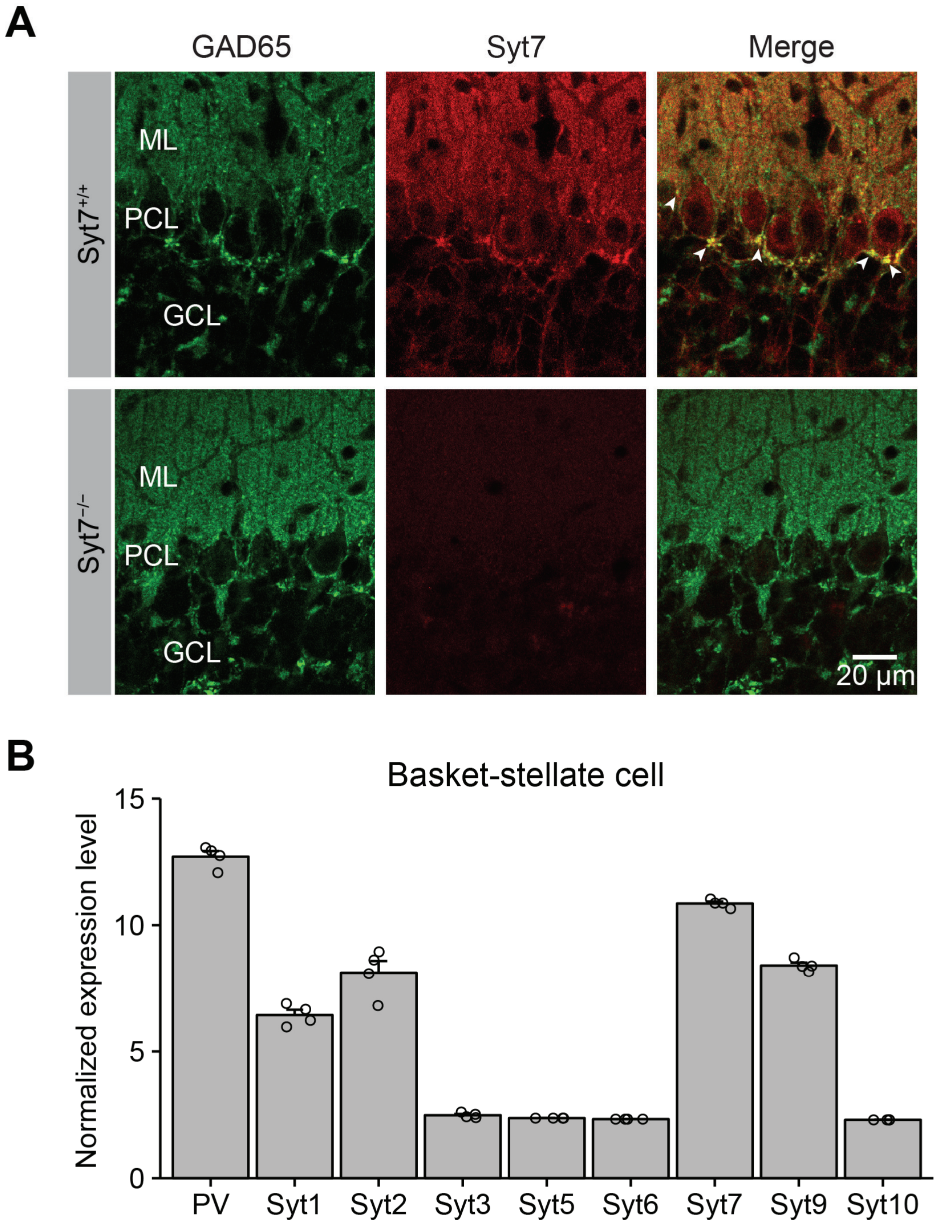

(**A**) Light-micrographs of cerebellar cortex, showing immunolabeling for GAD65 (left), Syt7 (center), and overlay (right) in $Syt7^{+/+}$ (top) and $Syt7^{-/-}$ (bottom) mice; single confocal sections. ML, molecular layer; PCL, Purkinje cell layer; GCL, granule cell layer. Note that Syt7 is highly abundant in GABAergic presynaptic terminals of cerebellar BCs surrounding somata of PCs.

(**B**) Expression level of different synaptotagmin isoforms in cerebellar basket-stellate interneurons. Only Ca^{2+}-binding synaptotagmin isoforms were depicted. Values were obtained by meta-analysis of the data from a previous microarray study (Paul et al., 2012). Expression levels were downloaded from gene expression omnibus (GEO; accession GSE37055; data from four mice) and normalized by GCRMA, as done in the original paper (Paul et al., 2012). Note that Syt7 is the most highly expressed synaptotagmin isoform in basket-stellate interneurons. PV, parvalbumin.

Figure S2. Deletion of Syt7 does not affect miniature IPSCs in PCs, related to Figure 1

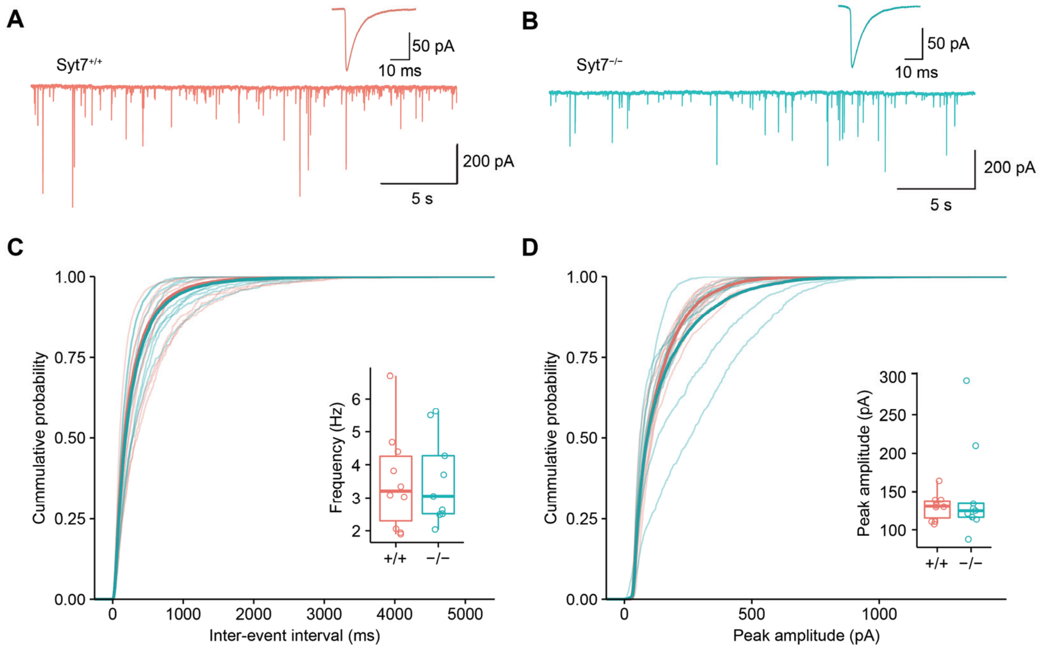

(**A** and **B**) Traces of miniature IPSCs at −70 mV in the presence of 1 μM TTX in $Syt7^{+/+}$ (A) and $Syt7^{-/-}$ mice (B). Insets show average miniature IPSCs from 1379 and 667 events, respectively.

(**C** and **D**) Cumulative distributions of miniature IPSC inter-event interval (C) and peak amplitude (D). Thin lines, distributions from individual cells; thick lines, average distributions. Data from 10 and 9 PCs, respectively. In box plots (insets in C and D), horizontal lines represent median; boxes, quartiles; whiskers, most extreme data points ≤1.5 interquartile range from box edges; and single points, data from individual experiments.

Figure S3. HdAd-mediated expression of Syt7 in Syt7$^{-/-}$ mice rescues the synaptic phenotype, related to Figure 2

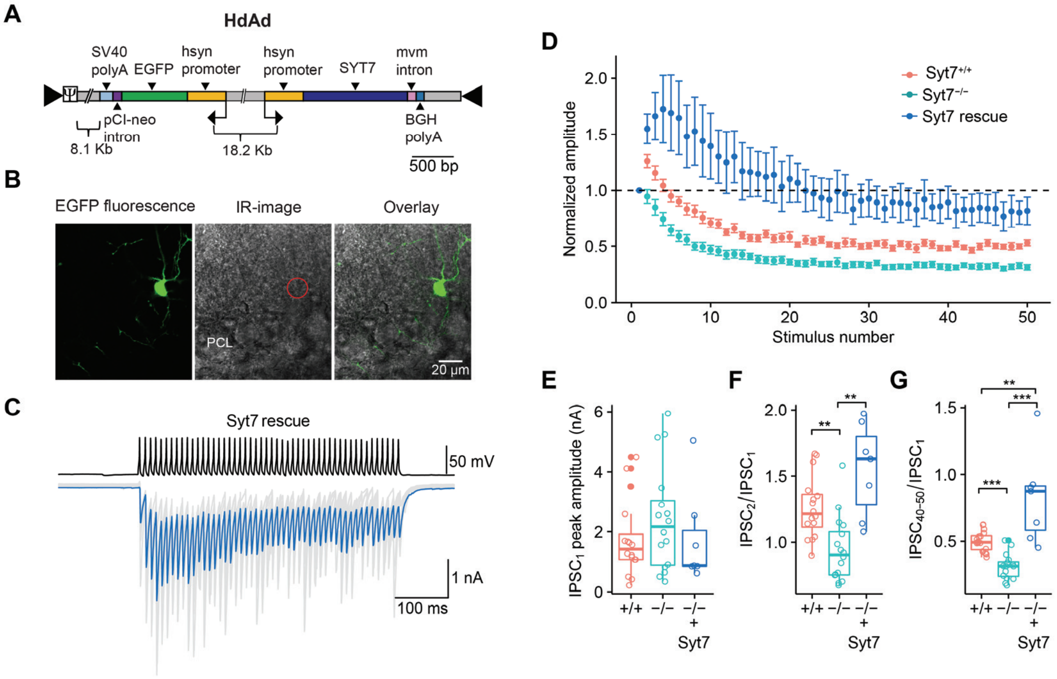

(**A**) Schematic illustration of the helper-dependent adenovirus (HdAd) constructs used for rescue experiments. Hsyn, human synapsin promoter; mvm, minute virus of mice intron; SV40 polyA, Simian virus 40 poly A; BGH polyA, bovine growth hormone poly A.

(**B**) Confocal microscopic image of a cerebellar BC in the molecular layer (left), infrared videomicroscopy light-micrograph (center), and overlay (right). The BC is highly fluorescent, showing successful infection with HdAd.

(**C**) IPSCs evoked by a 100-Hz train of 50 APs in Syt7$^{-/-}$ + HdAd-Syt7 synapses. Upper traces, presynaptic APs evoked by brief current pulses; lower traces, IPSCs (gray traces, individual sweeps; blue trace, average IPSC).

(**D**) Normalized IPSC peak amplitudes ($IPSC_n$ / $IPSC_1$), plotted against stimulus number (n). Red circles, Syt7$^{+/+}$; cyan circles, Syt7$^{-/-}$; blue circles, Syt7$^{-/-}$ + HdAd-Syt7. Syt7$^{-/-}$ + HdAd-Syt7 data were obtained from 7 pairs.

(**E–G**) Box plot of IPSC peak amplitude ($IPSC_1$; E), paired-pulse ratio ($IPSC_2$ / $IPSC_1$; F), and normalized steady-state IPSC amplitude ($IPSC_{40–50}$ / $IPSC_1$; G). Red circles, Syt7$^{+/+}$; cyan circles, Syt7$^{-/-}$; blue circles, Syt7$^{-/-}$ + HdAd-Syt7. ** in (F) indicates $P = 0.0014$, ** in (G) indicates $P = 0.008$, and *** in (G) represents $P < 0.001$.

In box plots, horizontal lines represent median; boxes, quartiles; whiskers, most extreme data points ≤1.5 interquartile range from box edges; and single points, data from individual experiments. Note that overexpression of Syt7 not only rescued the synaptic phenotype, but rather enhanced initial facilitation and steady-state IPSC amplitude values above control values, implying that exogenous Syt7 levels after HdAd infection in $Syt7^{-/-}$ mice were higher than endogenous Syt7 levels in $Syt7^{+/+}$ mice.

Figure S4. Syt7 ensures efficacy of high-frequency synaptic transmission in hippocampal BC–GC synapses, related to Figures 2 and 3

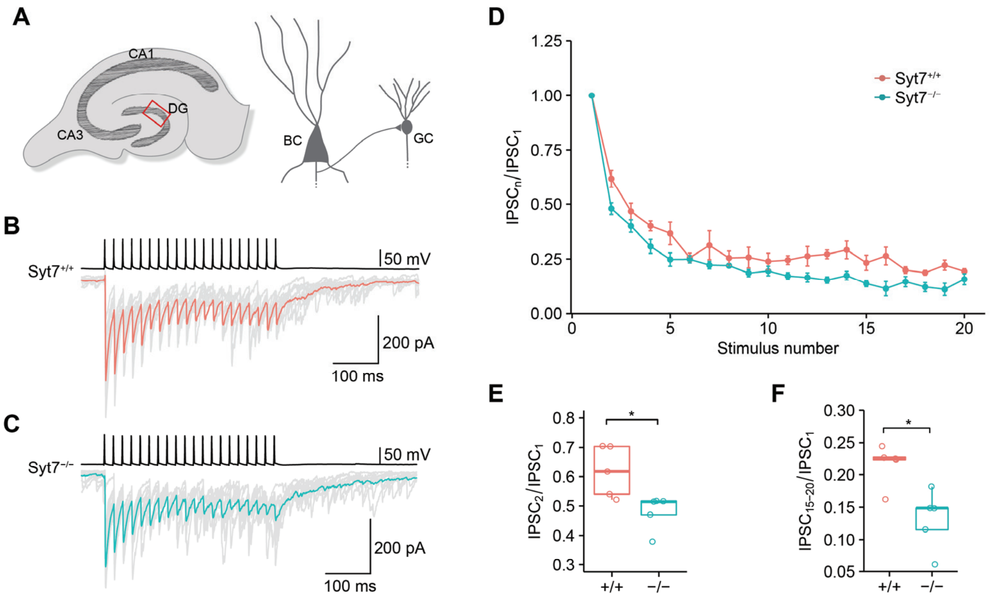

(**A**) Schematic illustration of the recording configuration in hippocampal slices. DG, dentate gyrus; CA3, cornu ammonis 3 subfield; CA1, cornu ammonis 1 subfield.

(**B** and **C**) IPSCs evoked by a 50-Hz train of 20 APs for Syt7$^{+/+}$ (B) and Syt7$^{-/-}$ synapses (C). Upper traces, presynaptic APs evoked by brief current pulses; lower traces, IPSCs (gray traces, individual sweeps; red and cyan traces, average IPSCs).

(**D**) Normalized IPSC peak amplitude, plotted against stimulus number. Red circles, Syt7$^{+/+}$ synapses; cyan circles, Syt7$^{-/-}$ synapses. Data were obtained from 5 pairs in each group.

(**E** and **F**) Box plot of paired-pulse ratio ($IPSC_2$ / $IPSC_1$; E) and normalized steady-state IPSC amplitude ($IPSC_{15–20}$ / $IPSC_1$; F). In box plots, horizontal lines represent median; boxes, quartiles; whiskers, most extreme data points ≤1.5 interquartile range from box edges; and single points, data from individual experiments. All experiments were performed in hippocampal BC–GC synapses. * indicates P = 0.01 in (E) and 0.02 in (F).

Table S1. Comparison of basic release properties of Syt7$^{+/+}$ and Syt7$^{–/–}$ synapses, related to Figure 1

**Parameter**	**Syt7$^{+/+}$**	**Syt7$^{–/–}$**	**P**
Latency [a]	0.66 ± 0.03 ms (15)	0.68 ± 0.03 ms (15)	0.756
SD of latency [a]	0.13 ± 0.01 ms (15)	0.10 ± 0.03 ms (15)	0.070
20%–80% rise time [a]	0.46 ± 0.02 ms (15)	0.58 ± 0.06 ms (15)	0.089
IPSC peak amplitude [a]	1.28 ± 0.26 nA (15)	1.54 ± 0.21 nA (15)	0.367
IPSC decay time constant [a]	8.45 ± 0.38 ms (15)	9.05 ± 0.48 ms (15)	0.372
IPSC half width [a]	7.40 ± 0.33 ms (15)	8.68 ± 0.61 ms (15)	0.120
Miniature IPSC frequency [b]	3.50 ± 0.47 Hz (10)	3.54 ± 0.44 Hz (9)	0.968
Miniature IPSC peak amplitude [b]	129.67 ± 5.38 pA (10)	147.89 ± 12.01 pA (9)	0.842
Frequency of asynchronous release [c]	5.57 ± 0.70 Hz (10)	3.51 ± 0.52 Hz (10)	0.034

Mean ± SEM (number of pairs / experiments).

[a] IPSCs evoked by single AP.

[b] Spontaneous IPSCs recorded in the presence of 1 μM TTX.

[c] IPSCs evoked by 20-Hz train of 20 APs.

Table S2. Comparison of synaptic dynamics of $Syt7^{+/+}$ and $Syt7^{–/–}$ synapses, related to Figures 2 and 4

Parameter [a]	$Syt7^{+/+}$	$Syt7^{–/–}$	P
$IPSC_2$ / $IPSC_1$ (10 Hz)	0.797 ± 0.037 (10)	0.838 ± 0.031 (10)	0.481
$IPSC_{15–20}$ / $IPSC_1$ (10 Hz)	0.527 ± 0.023 (10)	0.465 ± 0.020 (10)	0.089
$IPSC_2$ / $IPSC_1$ (20 Hz)	1.000 ± 0.087 (10)	0.837 ± 0.032 (10)	0.248
$IPSC_{15–20}$ / $IPSC_1$ (20 Hz)	0.567 ± 0.050 (10)	0.436 ± 0.013 (10)	0.043
$IPSC_2$ / $IPSC_1$ (50 Hz)	1.249 ± 0.063 (10)	0.904 ± 0.045 (10)	0.007
$IPSC_{15–20}$ / $IPSC_1$ (50 Hz)	0.608 ± 0.044 (10)	0.435 ± 0.023 (10)	0.003
$IPSC_2$ / $IPSC_1$ (100 Hz)	1.261 ± 0.058 (16)	0.945 ± 0.061 (16)	<0.001
$IPSC_{15–20}$ / $IPSC_1$ (100 Hz)	0.579 ± 0.030 (16)	0.380 ± 0.028 (16)	<0.001

Mean ± SEM (number of pairs).

[a] IPSCs evoked by trains of APs at indicated frequency
